# Predicting breast cancer risk using personal health data and machine learning models

**DOI:** 10.1371/journal.pone.0226765

**Published:** 2019-12-27

**Authors:** Gigi F. Stark, Gregory R. Hart, Bradley J. Nartowt, Jun Deng

**Affiliations:** Department of Therapeutic Radiology, Yale University, New Haven, CT, United States of America; Federal University of Technology - Paraná, BRAZIL

## Abstract

Among women, breast cancer is a leading cause of death. Breast cancer risk predictions can inform screening and preventative actions. Previous works found that adding inputs to the widely-used Gail model improved its ability to predict breast cancer risk. However, these models used simple statistical architectures and the additional inputs were derived from costly and / or invasive procedures. By contrast, we developed machine learning models that used highly accessible personal health data to predict five-year breast cancer risk. We created machine learning models using only the Gail model inputs and models using both Gail model inputs and additional personal health data relevant to breast cancer risk. For both sets of inputs, six machine learning models were trained and evaluated on the Prostate, Lung, Colorectal, and Ovarian Cancer Screening Trial data set. The area under the receiver operating characteristic curve metric quantified each model’s performance. Since this data set has a small percentage of positive breast cancer cases, we also reported sensitivity, specificity, and precision. We used Delong tests (*p* < 0.05) to compare the testing data set performance of each machine learning model to that of the Breast Cancer Risk Prediction Tool (BCRAT), an implementation of the Gail model. None of the machine learning models with only BCRAT inputs were significantly stronger than the BCRAT. However, the logistic regression, linear discriminant analysis, and neural network models with the broader set of inputs were all significantly stronger than the BCRAT. These results suggest that relative to the BCRAT, additional easy-to-obtain personal health inputs can improve five-year breast cancer risk prediction. Our models could be used as non-invasive and cost-effective risk stratification tools to increase early breast cancer detection and prevention, motivating both immediate actions like screening and long-term preventative measures such as hormone replacement therapy and chemoprevention.

## Introduction

Among women, breast cancer is the most frequently diagnosed cancer and a leading cause of death [1]. In the developed world, between 1 in 8 and 1 in 12 women will have breast cancer during her lifetime [2]. Predicting breast cancer risk is vital to combating this disease. There are two primary types of breast cancer risk [2]. The first type represents the probability that an individual will contract breast cancer during a specified period of time [2]. The second type reflects the probability that a mutation will occur in a high-risk gene [2]. We hope to develop a model that accurately predicts the former type of risk. Current breast cancer screening guidelines are limited. The United States Preventative Services Task Force recommends screening for women ages 50-74 and does not provide conclusive guidelines for women outside this age range [3]. Accurately predicting breast cancer risk can both encourage screening for high-risk women who otherwise would not be screened and promote adherence to screening guidelines among those who otherwise might not follow them. A statistical model that assesses breast cancer risk can also inform chemoprevention and other actions to diminish one’s risk [4]. Thus, the goal of this study is to develop models that effectively predict women’s five-year breast cancer risks.

Many previous works used the Gail model to assess breast cancer risk. The Gail model is a statistical model that estimates breast cancer risk for women with no personal history of the disease and no known mutations in high-risk genes [5]. This model traditionally has six self-reported inputs: current age, age at menarche, age at first live birth, number of first-degree relatives who have had breast cancer, race / ethnicity, and number of previous breast biopsies [5]. The Gail model incorporates both age-specific breast cancer hazard rates and these inputs (whose weights are determined by a logistic regression) to predict a woman’s breast cancer risk [6]. The Breast Cancer Risk Prediction Tool (BCRAT) is an implementation of the Gail model that makes use of data regarding personal history of atypical hyperplasia, if it is available, in addition to the traditional six Gail model inputs [7].

The Gail model, however, is far from perfect. This model has failed to effectively predict breast cancer risk for some population segments [8, 9]. Additionally, the model has had difficulty discriminating between breast cancer and non-breast cancer cases at the individual level [10]. One work found that a logistic regression with only age, number of previous breast biopsies, and family history of breast cancer as inputs performed comparably to the Gail model in predicting five-year breast cancer risk [9].

There is a plethora of previous research that demonstrates how the addition of costly inputs to the Gail model can improve its predictive power. Previous papers found that when validated on the same data set, models that were fed one or more informative inputs in addition to the Gail model inputs better predicted breast cancer risk than the Gail model itself. These works implemented simple statistical models and incorporated inputs derived from costly and / or invasive procedures. These inputs included breast density [11–13], genetic single-nucleotide polymorphism [12–16], nipple aspirate fluid cytology [17], and / or hormone level data [12, 18]. All of these works made use of simple statistical models such as Cox proportional hazards regressions [11, 17], logistic regressions [12, 16], or Gail model implementations [13–15, 18].

This study examines whether complex machine learning models can better predict five-year breast cancer risk than the BCRAT [7] does. Complex machine learning models can pick up on subtler patterns in input data and thus can be more effective predictors. Using machine learning models and personal health data, Hart et al. [19] effectively predicted endometrial cancer risk. Hart et al. [19] compared the endometrial cancer risk prediction of six machine learning algorithms: logistic regression, naive Bayes, decision tree, linear discriminant analysis, support vector machine, and feed-forward artificial neural network. They performed internal validation on the Prostate, Lung, Colorectal, and Ovarian Cancer Screening Trial (PLCO) data set [20] by training models on 70% of the data set and evaluating the performance of these models on the remaining 30%. Hart et al. [19] found that the neural network was the best risk predictor. We seek to determine whether breast cancer risk, like endometrial cancer risk, can be effectively predicted using machine learning models. By comparing the performance of various machine learning models to the performance of the BCRAT [7] when both models are fed identical inputs and evaluated on the same data set, we can determine whether a model with a stronger statistical foundation outperforms the BCRAT [7].

This study also analyzes whether models that are fed both the BCRAT [7] inputs and additional informative inputs have greater predictive power than the BCRAT alone has. These additional inputs, which include age at menopause, an indicator of current hormone usage, number of years of hormone usage, body mass index (BMI), pack years of cigarettes smoked, years of birth control usage, number of live births, and an indicator of personal prior history of cancer, are all relevant to breast cancer risk [21–24]. We evaluate these machine learning models and the BCRAT [7] on the same data set. By comparing the performance of these machine learning models to the performance of the BCRAT [7], we can determine whether the combination of additional inputs and more complex statistical models improves breast cancer risk prediction.

All inputs to our machine learning models can be easily and cheaply obtained from electronic health records. Thus, if stronger than the BCRAT [7], our models could provide the foundations for cost-effective and non-invasive tools to predict breast cancer risk. Women could use these tools to obtain initial breast cancer risk estimates before undergoing mammograms or genetic tests.

The goal of our research is to develop machine learning models that predict five-year breast cancer risk better than the BCRAT [7] does, and thus can be used to improve early detection and prevention of breast cancer. Given its success in predicting endometrial cancer risk [19], we hypothesize that the neural network will be the strongest machine learning model for both sets of inputs and the most likely to be more effective than the BCRAT [7]. This study is structured as follows. First, we detail how predictor variables were selected and processed. Then, we describe how our six machine learning classifiers were implemented, evaluated, and compared. Specifically, we examined the area under the receiver operating characteristic curve (AUC) performance of these machine learning models in using personal health data to predict five-year breast cancer risk. We compared the performance of the BCRAT [7] to the performance of each machine learning model when fed either BCRAT inputs or the broader set of inputs. Finally, we describe our results and their limitations and implications for breast cancer detection and prevention.

## Materials and methods

### Data

Models were trained and evaluated on the PLCO data set [20]. This data set was generated as part of a randomized, controlled, prospective study that sought to determine the effectiveness of different prostate, lung, colorectal, and ovarian cancer screenings. This trial was sponsored by the National Cancer Institute. From November 1993 to July 2001, participants enrolled in the study and filled out a baseline questionnaire detailing their previous and current health conditions. All processing of this data set was completed in Python (version 3.6.7) [25].

We initially downloaded data for all of the women in the PLCO data set. This data set consists of 78,215 women ages 50-78. We chose to exclude women who met any of the following conditions: 1) were missing data regarding whether they had been diagnosed with breast cancer and / or the timing of the diagnosis, 2) were diagnosed with breast cancer before the baseline questionnaire, 3) did not self-identify as White, Black, or Hispanic, 4) identified as Hispanic but did not have information available about where they were born, or 5) were missing data for any of the thirteen selected predictors. We excluded women who were diagnosed with breast cancer before the baseline questionnaire because the BCRAT [7] is not valid for women with a personal history of breast cancer. The BCRAT [7] is also not valid for women who have had chest radiation treatment or mutations in the BCRA1 or BCRA2 genes, or have a personal history of lobular carcinoma in situ, ductal carcinoma in situ, or other rare syndromes that tend to cause breast cancer such as Li-Fraumeni Syndrome. As data on these conditions was not available in the PLCO data set [20], we assumed that these conditions did not apply to any of the women in the data set. We excluded certain subjects based on self-identified race / ethnicity because only the PLCO [20] White, Black, and Hispanic race / ethnicity categories matched up with those of the BCRAT implementation [26] that we used. We did not include subjects who self-identified as Hispanic but did not have data available regarding their place of birth, because the BCRAT [7] implementation used different breast cancer composite incidences for United States-born and foreign-born Hispanic women [26]. In removing subjects based on these criteria, we reduced the pool to 64,739 women.

We trained a set of machine learning models that were fed five of the usual seven inputs to the BCRAT [7]. These five inputs, which included age, age at menarche, age at first live birth, number of first-degree relatives who have had breast cancer, and race / ethnicity, were the only traditional BCRAT [7] inputs available in the PLCO data set [20]. The BCRAT [7] to which we compared our machine learning models was also fed these five inputs.

Our inputs to the models with the broader set of predictors included the five BCRAT [7] inputs and eight additional factors. These additional predictors, selected based on availability in the PLCO data set [20] and relevance to breast cancer risk [21–24], included age at menopause, an indicator of current hormone usage, number of years of hormone usage, BMI, pack years of cigarettes smoked, years of birth control usage, number of live births, and an indicator of personal prior history of cancer.

We made a limited number of modifications to the predictor variables in order to facilitate the training and testing of our models. First, we appropriately assigned values to categorical variables. The PLCO data set presented the age at menarche, age at first live birth, age at menopause, years of hormone usage, and years of birth control usage data as categorical variables [20]. For example, the age at menarche variable was encoded to 1 for younger than 10 years old, 2 for 10 to 11 years old, 3 for 12 to 13 years old, 4 for 14 to 15 years old, and 5 for 16 years old and older. For values of categorical variables that represented a maximum number of years / an age or less (e.g., 10 years old and younger), we set our variable’s value to the maximum value (e.g., 10 years old). For values that represented a range strictly less than a maximum value (e.g., younger than 10 years old), we made the variable’s value equal the range’s upper limit (e.g., 10 years old). Similarly, for values that represented a minimum number of years / age or greater (e.g., 16 years old or older), we set the value to the minimum value (e.g., 16 years old). For values that encompassed a closed range (e.g., 12-13 years old), we set our variable’s value to the middle of the range (e.g., 12.5 years old).

After modifying the categorical variables, we made a few adjustments to the versions of the age at first live birth and the race / ethnicity variables fed into the machine learning models. For the BCRAT [7] model, we set the age at first live birth variable to 98 for nulliparous women (as the “BCRA” package (version 2.1) [26] in R (version 3.4.3) [27] used for implementing the BCRAT [7] indicated to do) and provided different race / ethnicity category values for the foreign-born and United States-born Hispanic women. For the machine learning models, we set the age at first live birth variable to current age for nulliparous women and represented race / ethnicity with two indicators, one for White women and one for Black women. Each woman was only categorized as one race / ethnicity (White, Black, or Hispanic). Thus, we did not need a Hispanic ethnicity indicator in addition to the White and Black race indicators. A Hispanic woman was one who had a 0 for both the White and Black race indicators. For the machine learning models, we did not distinguish between Hispanic women born in the United States and those born abroad.

Finally, we ensured that the versions of all variables fed into the machine learning models were scaled to lie between 0 and 1. Rescaling machine learning model inputs is important because doing so helps the models to train more quickly and to better generalize to all predictors [28]. We chose normalization by Eq (1) because it is one of the most popular methods of rescaling data [28]. In this equation, *x_i_* is the original *i^th^* value of the variable *x*, and *z_i_* is the rescaled value [28].

$$\begin{array}{c} z_{i}=\frac{x_{i}-min\left(x\right)}{max(x)-min(x)} \end{array}$$ (1)

For all models, the outcome variable indicated those who had breast cancer within five years (calculated as 1826 days) of their PLCO [20] baseline questionnaire. Out of 64,739 women, there were 1343 positive and 63,396 negative five-year breast cancer cases. Additional information on the breakdown of the predictor variables can be seen in Table 1.

**Table 1:** Input variables.

Input	Breast Cancer	Non-Breast Cancer
Age	62.6 (± 5.3)	62.5 (± 5.4)
Age at Menarche	12.6 (± 1.5)	12.7 (± 1.5)
Age at First Live Birth*	23.7 (± 4.6)	23.0 (± 4.3)
Number of First-Degree Relatives Who Have Had Breast Cancer	0.2 (± 0.5)	0.2 (± 0.4)
Race / Ethnicity		
White	94.8%	92.8%
Black	3.6%	5.6%
Hispanic (Born in US)	1.4%	1.3%
Hispanic (Born Abroad)	0.2%	0.3%
Age at Menopause	48.6 (± 5.0)	48.0 (± 5.1)
Current Hormone Usage	58.8%	50.6%
Years of Hormone Usage	4.7 (± 4.1)	4.1 (± 4.1)
BMI	27.1 (± 5.3)	27.3 (± 5.6)
Pack Years of Cigarettes Smoked	14.6 (± 23.9)	13.3 (± 22.4)
Birth Control Usage	2.9 (± 3.6)	2.7 (± 3.6)
Number of Live Births	2.8 (± 1.4)	2.9 (± 1.5)
Personal History of Prior Cancer	4.2%	3.4%

We show the means and standard deviations for the continuous variables and the percentages for the indicators.

* Among parous women.

We randomly split the PLCO data set [20] into 80% training data (1074 breast cancer and 50,717 non-breast cancer cases) and 20% testing data (269 breast cancer and 12,679 non-breast cancer cases) with essentially equal proportions of breast cancer cases in each set. All machine learning models were trained on the same training data set of subjects. The BCRAT [7] and all machine learning models were evaluated on the same testing data set of subjects. This type of testing qualifies as Transparent Reporting of a multivariable prediction model for Individual Prognosis Or Diagnosis (TRIPOD) level 2a validation [29].

### “The Breast Cancer Risk Prediction Tool”

We implemented the BCRAT [7] using the R programming language’s (version 3.4.3) [27] “BCRA” package [26]. For each individual, we set the values for the number of previous breast biopsies and for the indicator of atypical hyperplasia in a biopsy to 99, which reflects that these values are “unknown”. We ensured that the BCRAT [7] predicted five-year risk. We used the “BCRA” package’s “absolute.risk” function [26] to calculate BCRAT [7] risk predictions.

### Six types of machine learning models

We evaluated the effectiveness of six machine learning binary classifiers in predicting the probabilities that individuals develop breast cancer during the five years following their PLCO [20] baseline questionnaire. Our six types of classifiers were: logistic regression, Gaussian naive Bayes, decision tree, linear discriminant analysis, support vector machine, and feed-forward artificial neural network. We selected these machine learning models because each holds significant advantages that could make it the best model for predicting five-year breast cancer risk based on the given inputs.

The logistic regressions classify data by using maximum likelihood functions to predict the probabilities of outcome classes [30]. Logistic regressions are widely-used, simple, interpretable, and make no assumptions about the distribution of the explanatory data [31]. However, logistic regressions lack statistical sophistication, as they assume that inputs are linearly related to the log odds of the outcome [32]. In order for logistic regressions to model nonlinear relationships between variables, the model developer must search for these relationships before training and at times perform sophisticated transformations of variables [32].

Naive Bayes models are probabilistic classifiers [30]. They are constructed using the Bayes Theorem of conditional probabilities [30]. Naive Bayes models generally require less training data than do other classifiers and have fewer parameters than do models such as neural networks and support vector machines [33]. Naive Bayes models also do a good job of disregarding irrelevant inputs and noise [33]. However, naive Bayes models assume that input variables are independent, which is rarely true in classification tasks [30]. Despite this assumption, naive Bayes models have proven successful for a variety of tasks [30].

Decision trees classify data by posing recursive questions about predictor variables [30]. Decision trees are made up of nodes (which represent a test for a particular input), branches (which represent responses to node tests), and leaves (which are nodes at the bottom of the tree that provide ultimate classifications) [34]. Decision trees are highly interpretable, as the steps to obtain each subject’s classification can be easily comprehended [30]. Such an interpretable model could be preferable in a clinical setting.

Linear discriminant analysis models determine a linear decision boundary between classes [31] by maximizing the ratio of between-class variance to within-class variance [35]. Linear discriminant analysis takes advantage of dimensionality reduction techniques, transforming data into a lower dimensional space when making classifications [35]. One disadvantage of linear discriminant analysis models is that they assume that predictors are normally distributed, which they often are not, leading to poor performance [31].

Support vector machines, first presented in Cortes & Vapnik [36], find the hyperplane in the space of the predictors that maximizes the distance between the points corresponding to training data set subjects of different output classes [34]. Support vector machines generalize to different data sets well and work effectively with high dimensional data [30]. However, they are hard to interpret and require a lot of parameter tuning [30].

Neural networks are models loosely based on the structure of the brain [30]. These models are made up of layers of neurons that relate to each other via weighted connections [30]. These weights are adjusted during training through a process called backpropagation [30]. Neural networks can handle noisy data and model complex nonlinear functions well [30]. Neural networks, however, require a lot of parameter tuning [30], are difficult to interpret, and are subject to overfitting [32].

The Python programming language (version 3.6.7) [25] was used to generate all machine learning model code. We implemented the logistic regression, naive Bayes, decision tree, support vector machine, and linear discriminant analysis models using the Python scikit-learn package (version 0.20.1) [37]. For the logistic regression, we used the “linear_model.LogisticRegression” function. For the naive Bayes, our function was “naive_bayes.GaussianNB”. The “tree.DecisionTree” function was used to create a decision tree. For the support vector machine, we used the “svm.SVC” implementation with probability predictions enabled. For the linear discriminant analysis, we used a “discriminant_analysis.LinearDiscriminantAnalysis” model. For each of these functions, we used the relevant package’s default parameters.

We developed our neural networks using Python’s TensorFlow (version 1.8.0) [38]. All neural network weights were Xavier initialized, and all biases were initialized as constants. All neural networks had logistic activation functions after each hidden layer and after the output layer. We used cross-entropy to define loss and an Adam optimizer to minimize this loss.

### Evaluating and comparing model performance

For the BCRAT [7] model and all machine learning models, we reported the testing data set AUC, otherwise known as the concordance statistic, and the Delong [39] 95% confidence interval of this statistic. We used AUC to evaluate our models because many previous breast cancer risk prediction papers used this metric [11–18]. We used a Delong [39] method to calculate the 95% confidence interval of each AUC because this method is a nonparametric approach that makes fewer assumptions than other approaches do. AUCs and confidence intervals of AUCs were calculated in the R programming language (version 3.4.3) [27] due to the availability of the “pROC” [40] package in R. AUCs were calculated using the “pROC” [40] package’s “roc” function. We ensured that all AUCs were calculated in the correct direction by altering the direction for any models that produced warnings. Confidence intervals were measured by the “pROC” [40] package’s “ci.auc” function.

We determined the appropriate hyperparameters for the neural networks through 10-fold cross-validation on the training portion of the PLCO data set [20]. We tested a variety of hyperparameters for the neural networks. For both the models with only BCRAT [7] predictors and the models with the broader set of predictors, we experimented with 1 and 2 hidden layers, learning rates of 0.001, 0.005, and 0.01, and 2500, 5000, 7500, and 10,000 steps of backpropagation. For the neural network with only BCRAT [7] predictors, we examined models with 4 and 6 neurons per hidden layer. For the neural network with the broader set of predictors, we looked at models with 4, 6, 8, 10, 12, and 14 neurons per hidden layer. For these ranges of parameters, we evaluated every combination of values for number of hidden layers, number of neurons per hidden layer, learning rate, and number of steps of backpropagation. For each combination of hyperparameters, we ran 10 iterations of 10-fold cross-validation neural networks. For each fold in each iteration, we trained a model on 90% of the data and evaluated the model on the remaining 10%. For each iteration, we then calculated an AUC (using R’s “pROC” [40] package) based on all of the folds’ labels and predictions. For each set of inputs and combination of hyperparameters, we determined the mean and standard deviation of AUC across all 10 iterations. The neural network hyperparameters used for the model trained on the entire training data set and evaluated on the testing set corresponded to the best output (highest mean minus standard deviation AUC) across 10 iterations of 10-fold cross-validation neural networks.

As our classification problem is unbalanced (roughly 2% positive cases), we report each machine learning model’s sensitivity (otherwise known as recall), specificity, and precision in addition to the model’s AUC. Breast cancer data sets are inherently unbalanced as population breast cancer prevalence (in the United States in 2016), like the PLCO data set five-year breast cancer prevalence, is roughly 2% [41, 42]. Sensitivity indicates the ability of a model to label positive breast cancer cases as positive, whereas specificity marks the ability of a model to identify negative breast cancer cases as negative. High values for these variables indicate that a model predicts risk well. Precision indicates the likelihood that a woman actually has breast cancer when the model predicts that she has it. In other words, precision reflects the ability of a model to target only actual positive cases.

We calculated these metrics by first obtaining risk predictions for the training data set subjects from the Python machine learning models. All further calculations of these metrics were completed in R due to the availability of the “pROC” [40] package in this language. We used the “pROC” package’s [40] “roc” function to generate a “roc object” from the training data set labels and risk predictions. We then fed the “pROC” “coords” function this “roc object” so it could compute the threshold that maximized the sum of training data set sensitivities and specificities. We used this threshold to classify testing data set subjects as positive or negative cases based on their risk predictions. Using the actual test labels and these binary test predictions, we calculated the number of true positive, true negative, false positive, and false negative cases. We set sensitivity to the number of true positives divided by the sum of the number of true positives and the number of false negatives and set specificity to the number of true negatives divided by the sum of the number of true negatives and the number of false positives. Since the data set has relatively few five-year breast cancer cases and is thus biased, we calculated precision from sensitivity, specificity, and breast cancer prevalence (see Eq (2)). We calculated prevalence as the estimated number of women in the United States living with breast cancer divided by the estimated number of women living in the United States. Breast cancer incidence was based on National Cancer Institute Surveillance, Epidemiology, and End Results Program 2016 data [41]. We used 2016 breast cancer incidence because this is the most recent incidence reported by this program. Accordingly, we used 2016 female population estimates recorded by the United States Census Bureau [42].

$$\begin{array}{c} \text{precision}=\frac{\text{sensitivity}\;*\;\text{prevalence}}{\text{sensitivity}*\text{prevalence}+(1-\text{specificity})*(1-\text{prevalence})} \end{array}$$ (2)

Our examined BCRAT [7], implemented via the “BCRA” R package [26], was a pretrained model, and thus we did not have access to its training data. Since the BCRAT’s [7] training data was not available, we could not calculate sensitivity and specificity, and thus precision by identifying the threshold that maximized the sum of training data set sensitivities and specificities. Instead, we reported BCRAT [7] sensitivity, specificity, and precision values calculated using the threshold that maximized the sum of testing data set sensitivities and specificities. All other steps for calculating these BCRAT [7] metrics were identical to the steps taken for the machine learning model metrics. We could not make a fair direct comparison between these BCRAT [7] metrics and the corresponding machine learning model metrics as the thresholds used to calculate sensitivity and specificity values were based on different data sets. Rather we sought to examine whether the BCRAT [7] and machine learning model metrics lay in the same general ranges so we could put the machine learning model values into context. Using testing data set thresholds rather than training data set thresholds should boost sensitivity, specificity, and precision values. Thus, if BCRAT [7] metric values were generally comparable to the corresponding machine learning model metric values, then the machine learning models seemingly did not handle the unbalanced nature of this problem any worse than the BCRAT [7] did.

Since the Delong paper [39] did not indicate any particular method for calculating confidence intervals for sensitivity, specificity, and precision, we approximated asymptotic confidence intervals for these metrics. We reported Ward 95% binomial confidence intervals for sensitivity and specificity. Where $\hat{p}$ is the examined statistic (sensitivity or specificity), the confidence interval was ($\hat{p}-1.96$ $\sqrt{\hat{p}\left(1-p\right)/n}$, $\hat{p}+1.96$ $\sqrt{\hat{p}\left(1-p\right)/n}$). In this equation, *n* was the number of breast cancer cases for sensitivity and the number of non-breast cancer cases for specificity. In order to take into account its dependencies on both sensitivity and specificity, we calculated Steinberg [43] 95% confidence intervals for precision. The precision confidence intervals were calculated per Eqs (3), (4), (5), (6), (7), (8), (9), and (10):

ϕ=log(1-specificity)-log(sensitivity) (3)

P=proportionoffive-yearbreastcancersubjectsintestingdata (4)

Q=1-P=proportionofnonfive-yearbreastcancersubjectsintestingdata (5)

$$\sigma =\sqrt{\frac{1-\text{sensitivity}}{\text{sensitivity}*P}+\frac{\text{specificity}}{(1-\text{specificity})*Q}}$$ (6)

$$L=\phi -1.96*\sigma /\sqrt{n}$$ (7)

$$U=\phi +1.96*\sigma /\sqrt{n}$$ (8)

$$\text{Confidence}\;\text{Interval}\;\text{Lower}\;\text{Limit}=\frac{1}{1+\frac{exp(U)*(1-\text{prevalence})}{\text{prevalence}}}$$ (9)

$$\text{Confidence}\;\text{Interval}\;\text{Upper}\;\text{Limit}=\frac{1}{1+\frac{exp(L)*(1-\text{prevalence})}{\text{prevalence}}}$$ (10)

We compared the performance of two models using a Delong [39] test. We used a Delong [39] test to compare models because it is a nonparametric approach that makes fewer assumptions than other approaches. We implemented the Delong [39] test through the “pROC” [40] package’s “roc.test” function. We classified the difference between two models as statistically significant if the comparison had a two-sided *p*-value less than 0.05. For each set of input predictors, we compared the performance of each of the machine learning models to that of the BCRAT [7]. Also for each set of input predictors, we compared the model(s) with the highest AUC to all other models. The top models were the model(s) with the highest AUC and any models that were not statistically significantly weaker than the model(s) with the highest AUC.

For each set of input predictors, we plotted the receiver operating characteristic (ROC) curves of the BCRAT [7] and of each machine learning model. These curves had sensitivity (true positive rate) on the y-axis and specificity (true negative rate) on the x-axis. We generated these plots in R [27] because all model statistics were computed in this language.

## Results

For both sets of inputs, the selected neural network hyperparameters were those that produced the highest mean minus standard deviation AUC across 10 iterations of neural networks trained using 10-fold cross-validation on the training data set. For neural networks with only BCRAT [7] inputs, these hyperparameters were 6 neurons per hidden layer, 2 hidden layers, an 0.01 learning rate, and 5000 steps of backpropagation. For the neural networks with a broader set of inputs, the hyperparameters were 12 neurons per hidden layer, 2 hidden layers, an 0.01 learning rate, and 2500 steps of backpropagation.

**Fig 1:**
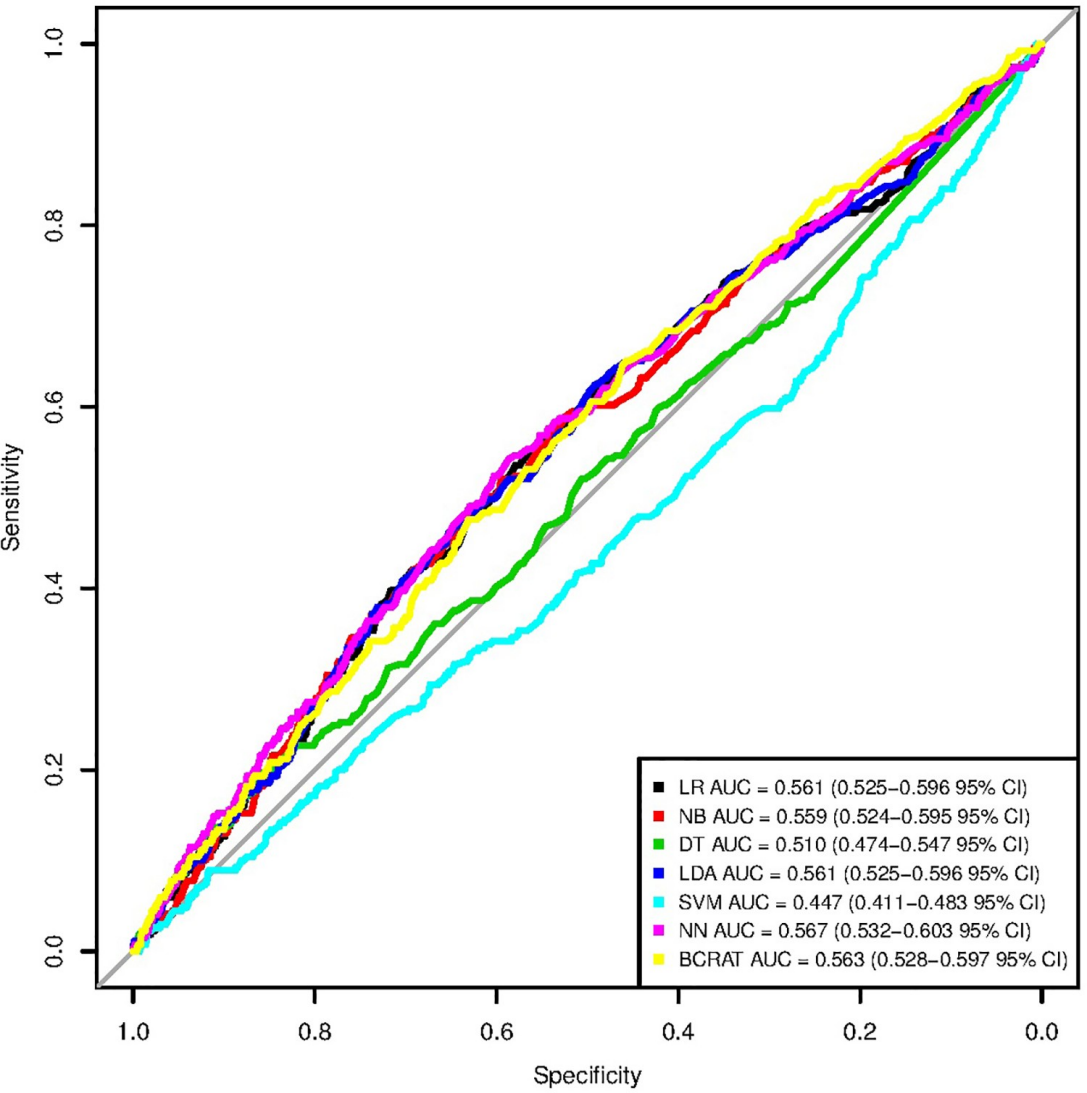
ROC curves for machine learning models with only BCRAT inputs and for the BCRAT. These are the receiver operating characteristic (ROC) curves for the six machine learning models with only Breast Cancer Risk Prediction Tool (BCRAT) inputs and for the BCRAT. The six machine learning models include a logistic regression (LR), naive Bayes (NB), decision tree (DT), linear discriminant analysis (LDA), support vector machine (SVM), and neural network (NN). We report Delong 95% confidence intervals for each area under the receiver operating characteristic curve (AUC) value.

None of the machine learning models with only BCRAT [7] predictors were significantly stronger than the BCRAT [7] (see Tables 2 and 3). The neural network, the model using only BCRAT [7] predictors with the highest AUC, was significantly stronger than the decision tree and the support vector machine models, but not significantly stronger than the logistic regression, linear discriminant analysis, or naive Bayes models (see Table 4). The ROC curves for each machine learning model and for the BCRAT [7] can be seen in Fig 1. The logistic regression, naive Bayes, linear discriminant analysis, and neural network models all had sensitivity values that were approximately 0.6 (values ranged from 0.587 for the linear discriminant analysis to 0.621 for the neural network). The specificities of these models were all around 0.5 (values ranged from 0.474 for the neural network to 0.512 for the linear discriminant analysis). The precisions of these models were all relatively low, hovering around 0.025. Despite using testing data set rather than training data set thresholds, the BCRAT [7] sensitivity, specificity, and precision values generally lay in the same ranges as the corresponding machine learning model metrics did.

**Table 2:** Statistics for machine learning models with only BCRAT inputs and for the BCRAT.

	AUC	Sensitivity	Specificity	Precision
**LR**	0.561 (0.525-0.596 95% CI)	0.599 (0.540-0.657 95% CI)	0.509 (0.501-0.518 95% CI)	0.0258 (0.0234-0.0284 95% CI)
**NB**	0.559 (0.524-0.595 95% CI)	0.602 (0.544-0.661 95% CI)	0.504 (0.496-0.513 95% CI)	0.0257 (0.0233-0.0282 95% CI)
**DT**	0.510 (0.474-0.547 95% CI)	0.387 (0.328-0.445 95% CI)	0.616 (0.607-0.624 95% CI)	0.0213 (0.0184-0.0248 95% CI)
**LDA**	0.561 (0.525-0.596 95% CI)	0.587 (0.529-0.646 95% CI)	0.512 (0.503-0.521 95% CI)	0.0254 (0.0230-0.0281 95% CI)
**SVM**	0.447 (0.411-0.483 95% CI)	0.993 (0.982-1.00 95% CI)	0.00718 (0.00571-0.00865 95% CI)	0.0212 (0.0210-0.0214 95% CI)
**NN**	0.567 (0.532-0.603 95% CI)	0.621 (0.563-0.679 95% CI)	0.474 (0.465-0.482 95% CI)	0.0249 (0.0227-0.0273 95% CI)
**BCRAT**	0.563 (0.528-0.597 95% CI)	0.647 (0.590-0.704 95% CI)*	0.461 (0.452-0.470 95% CI)*	0.0254 (0.0232-0.0277 95% CI)*

**Abbreviations**: CI = confidence interval, LR = logistic regression, NB = naive Bayes, DT = decision tree, LDA = linear discriminant analysis, SVM = support vector machine, NN = neural network

* Calculated using sensitivity / specificity values based on the threshold that maximized the sum of testing data set sensitivities and specificities rather than the sum of training data set sensitivities and specificities.

**Table 3:** Comparisons between machine learning models with only BCRAT inputs and the BCRAT.

	LR	NB	DT	LDA	SVM	NN
***z*-Value**	0.169	0.278	2.17	0.175	3.61	0.416
***p*-Value**	0.866	0.781	0.0302	0.861	3.08e-4	0.678

**Table 4:** Comparisons between machine learning models with only BCRAT inputs.

	NN / LR	NN / NB	NN / DT	NN / LDA	NN / SVM
***z*-Value**	0.888	1.07	2.40	1.08	3.58
***p*-Value**	0.375	0.286	0.0163	0.278	3.47e-4

**Fig 2:**
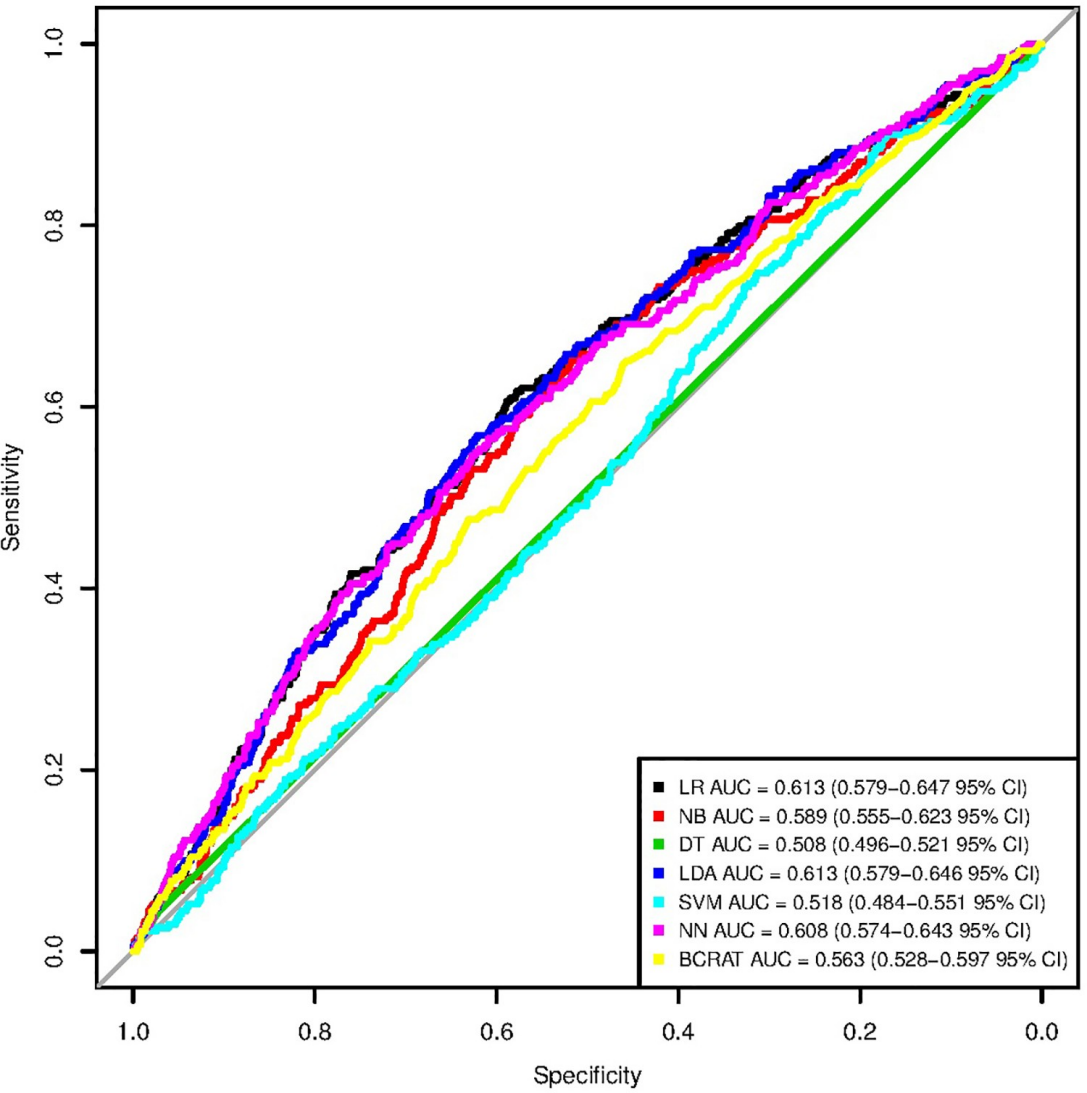
ROC curves for machine learning models with the broader set of inputs and for the BCRAT. These are the receiver operating characteristic (ROC) curves for the six machine learning models with the broader set of inputs and for the Breast Cancer Risk Prediction Tool (BCRAT). The six machine learning models include a logistic regression (LR), naive Bayes (NB), decision tree (DT), linear discriminant analysis (LDA), support vector machine (SVM), and neural network (NN). We report Delong 95% confidence intervals for each area under the receiver operating characteristic curve (AUC) value.

The logistic regression, linear discriminant analysis, and neural network models using the broader set of predictors were each significantly stronger than the BCRAT [7] (see Tables 5 and 6). The logistic regression and linear discriminant analysis models tied for the highest testing data set AUC (0.613). The logistic regression was significantly stronger than the decision tree, support vector machine, and naive Bayes models, but not stronger than the linear discriminant analysis or the neural network models. Similarly, the linear discriminant analysis was significantly stronger than the decision tree, support vector machine, and naive Bayes models, but not stronger than the logistic regression or neural network models (see Table 7). The ROC curves for each machine learning model and for the BCRAT [7] can be seen in Fig 2. The logistic regression, linear discriminant analysis, and neural network models had very different values for sensitivity. The logistic regression had a low sensitivity of 0.476, whereas the linear discriminant analysis had a sensitivity of 0.688. The neural network sensitivity lay between these two values at 0.599. For specificity, the logistic regression had the highest value at 0.691, followed by the neural network (0.562), and the linear discriminant analysis (0.467). The precisions for all three of these models were low. The logistic regression had a precision of 0.0323, slightly higher than the neural network (0.0287) and linear discriminant analysis (0.0272) precisions. Again, we saw that the machine learning model sensitivities, specificities, and precisions were generally comparable to those of the BCRAT [7].

**Table 5:** Statistics for machine learning models with the broader set of inputs and for the BCRAT.

	AUC	Sensitivity	Specificity	Precision
**LR**	0.613 (0.579-0.647 95% CI)	0.476 (0.416-0.536 95% CI)	0.691 (0.683-0.699 95% CI)	0.0323 (0.0285-0.0366 95% CI)
**NB**	0.589 (0.555-0.623 95% CI)	0.639 (0.582-0.697 95% CI)	0.523 (0.514-0.531 95% CI)	0.0282 (0.0258-0.0308 95% CI)
**DT**	0.508 (0.496-0.521 95% CI)	0.0446 (0.0199-0.0693 95% CI)	0.972 (0.969-0.974 95% CI)	0.0328 (0.0190-0.0562 95% CI)
**LDA**	0.613 (0.579-0.646 95% CI)	0.688 (0.632-0.743 95% CI)	0.467 (0.459-0.476 95% CI)	0.0272 (0.0251-0.0295 95% CI)
**SVM**	0.518 (0.484-0.551 95% CI)	0.517 (0.457-0.576 95% CI)	0.478 (0.469-0.486 95% CI)	0.0210 (0.0187-0.0235 95% CI)
**NN**	0.608 (0.574-0.643 95% CI)	0.599 (0.540-0.657 95% CI)	0.562 (0.553-0.570 95% CI)	0.0287 (0.0261-0.0317 95% CI)
**BCRAT**	0.563 (0.528-0.597 95% CI)	0.647 (0.590-0.704 95% CI)*	0.461 (0.452-0.470 95% CI)*	0.0254 (0.0232-0.0277 95% CI)*

* Calculated using sensitivity / specificity values based on the threshold that maximized the sum of testing data set sensitivities and specificities rather than the sum of training data set sensitivities and specificities.

**Table 6:** Comparisons between machine learning models with the broader set of inputs and the BCRAT.

	LR	NB	DT	LDA	SVM	NN
***z*-Value**	2.50	1.31	2.99	2.62	1.86	2.50
***p*-Value**	0.0123	0.189	2.77e-3	8.74e-3	0.0632	0.0123

**Table 7:** Comparisons between machine learning models with the broader set of inputs.

	LR / NB	LR / DT	LR / LDA	LR / SVM	LR / NN	LDA / NB	LDA / DT	LDA / SVM	LDA / NN
***z*-Value**	2.39	5.79	0.110	4.35	0.529	2.54	5.81	4.23	0.496
***p*-Value**	0.0168	6.85e-9	0.912	1.39e-5	0.597	0.0112	6.19e-9	2.35e-5	0.620

## Discussion

### Comparing machine learning models to the BCRAT

Comparisons between our machine learning models with the broader set of inputs and the BCRAT [7] suggest that a model that incorporates both the BCRAT inputs and additional easy-to-obtain personal health inputs can be an effective predictor of breast cancer risk. At an 0.05 level, the logistic regression, linear discriminant analysis, and neural network with the broader set of inputs were all significantly stronger than the BCRAT [7]. The effectiveness of these models indicates that they could be incorporated into risk stratification tools. Such tools could increase early breast cancer detection by prompting screening and could help reduce the incidence of breast cancer by motivating preventative actions.

We did not perform rigorous statistical comparisons between the BCRAT [7] and machine learning model sensitivity, specificity, and precision values as the BCRAT [7] metrics were derived from testing data set thresholds whereas the machine learning models made use of training data set thresholds. However, for both sets of inputs, the BCRAT [7] metrics were generally comparable to the machine learning model metrics. For example, the BCRAT [7] and the machine learning models all tended to have similarly low precision values. These results suggest that despite being trained on an unbalanced data set, these machine learning models did not handle the issue of imbalance worse than the BCRAT [7] did.

### Comparing machine learning models to each other

We hypothesized that the neural network would be the best machine learning model for both sets of inputs. Unexpectedly, when only BCRAT [7] inputs were used, the neural network was not statistically significantly stronger than the logistic regression, naive Bayes, or linear discriminant analysis models. When the broader set of inputs was used, the logistic regression and linear discriminant analysis models produced higher AUCs than the neural network did. However, these higher AUCs were not significantly stronger than that of the neural network. Perhaps the shortcomings of the neural networks for both sets of inputs were due to the limited size of the training data set or the selection of hyperparameters. The hyperparameters that produced the best results for iterations of 10-fold cross-validation neural networks across the training data set may not be best for the neural networks trained on the entire training set and evaluated on the testing data set. Perhaps the neural networks were not stronger than the logistic regression or linear discriminant analysis models because a simple linear decision boundary, which both of these models draw [31], is best for these tasks. When fed the broader set of inputs, a logistic regression, a model that is not more statistically complex than the BCRAT [7], is statistically significantly stronger than the BCRAT and is one of the models with the highest AUC. This result suggests that the incorporation of additional inputs, not the implementation of more complex machine learning models, has led to improved breast cancer risk prediction.

Among machine learning models with only BCRAT [7] inputs, it seems like all of the strongest models had larger sensitivities than specificities. For models that predict breast cancer risk, sensitivity, or the ability to detect positive cases, is more important than specificity, the ability to detect negative cases. The models’ relatively large sensitivities highlight their potential, even though they are not significantly stronger than the BCRAT [7].

Among the top machine learning models fed the broader set of inputs, the linear discriminant analysis had by far the highest sensitivity, but the lowest specificity. On the other hand, the logistic regression had a very low sensitivity and a high specificity. The neural network had a moderately high sensitivity and specificity. The importance of sensitivity highlights the value of the linear discriminant analysis model and undercuts the potential of the logistic regression.

### Limitations

One limitation was that models were trained and tested on separate portions of the same data set. Ideally, models would be trained on one data set and validated on another data set from a separate study. Such external validation can prove the generalizability of models. We did not have access to another data set that we could use to model prospective five-year breast cancer risk and thus were forced to split the PLCO data set [20] into training and testing portions. Further work could examine whether, if when trained on the entire PLCO data set, these machine learning models generalize well to new data sets. Future studies could also determine if logistic regression, linear discriminant analysis, and neural network models with our selected inputs predict breast cancer risk even better when trained on a larger data set.

Another potential limitation was the lack of available biopsy or atypical hyperplasia data in the PLCO data set [20]. It is possible that if all models had this data as available, then the BCRAT [7] would perform better than the logistic regression, linear discriminant analysis, or neural network models with the broader set of inputs. However, there is no reason to think that this information would not boost all models. Future studies could compare machine learning models to the BCRAT [7] when all models are fed all seven traditional BCRAT inputs.

### Potential applications

As our logistic regression, linear discriminant analysis, and neural network models with the broader set of inputs effectively predicted five-year breast cancer risk, these models could be used to inform and guide screening and preventative measures. Our models could easily be incorporated into phone application or website breast cancer risk prediction tools. Using our models as such would be convenient and cost-effective as our personal health data inputs are easy and inexpensive to obtain from electronic health records or an office visit. While the purpose of such a tool would not be to replace physician advice or mammograms, risk predictions derived from our models could contribute to both early breast cancer detection and breast cancer prevention. Women who receive high estimated risks could be motivated to seek out a doctor or take other preventative actions. Models could be used to guide immediate personal decisions such as screening. Doctors could also use these models to inform decisions on whether and when to recommend long-term preventative actions such as chemoprevention and hormone replacement therapy.

## Conclusion

Our logistic regression, linear discriminant analysis, and neural network models with the broader set of inputs predicted five-year breast cancer risk better than the BCRAT did. Whereas previous works have demonstrated that inputs derived from costly and / or invasive procedures can improve the Gail model’s performance, these results suggest that additional easy-to-obtain inputs can also improve the Gail model’s ability to predict breast cancer risk. Our models could serve as the bases of new cost-effective and non-invasive tools to inform and prompt screening and immediate and long-term preventative actions with the potential to increase early detection and reduce the incidence of breast cancer.
